# Nonadaptive molecular evolution of plastome during the speciation of *Actaea purpurea* and its relatives

**DOI:** 10.1002/ece3.9321

**Published:** 2022-09-17

**Authors:** Dan‐Qing Zhang, Yi Ren, Jian‐Qiang Zhang

**Affiliations:** ^1^ National Engineering Laboratory for Resource Development of Endangered Crude Drugs in Northwest China College of Life Sciences, Shaanxi Normal University Xi'an China; ^2^ Key Laboratory of Medicinal Plant Resource and Natural Pharmaceutical Chemistry of Ministry of Education Shaanxi Normal University Xi'an China

**Keywords:** *Actaea*, adaptive evolution, dN/dS, McDonald‐Kreitman test, plastome, speciation

## Abstract

We have seen an explosive increase of plant plastid genome (plastome) sequences in the last decade, and the view that sequence variation in plastomes is maintained by the mutation‐drift balance has been challenged by new evidence. Although comparative genomic and population‐level studies provided us with evidence for positive evolution of plastid genes at both the macro‐ and micro‐evolution levels, less studies have systematically investigated how plastomes have evolved during the speciation process. We here sequenced 13 plastomes of *Actaea purpurea* (P.K. Hsiao) J. Compton, and its closest relatives, and conducted a systematic survey of positive selection in their plastid genes using the McDonald‐Kreitman test and codon‐based methods using maximum likelihood to estimate the ratio of nonsynonymous to synonymous substitutions (*ω*) across a phylogeny. We found that during the speciation of *A. purpurea* and its relatives, all plastid genes evolved neutrally or were under purifying selection. Genome size, gene order, and number were highly conserved. Comparing to *A. purpurea*, plastomes of *Actaea japonica* and *Actaea biternata* had low genetic diversity, consistent with previous studies. Our work not only sheds important light on the evolutionary history of *A. purpurea* and its kin, but also on the evolution of plastomes during plant speciation.

## Graphical abstract

Thirteen plastomes of 1representative populations of *Actaea purpurea* and its relatives were conserved with respect to size, structure, gene content, and order, with few variations. Positive selection analysis suggests that all plastid genes evolve neutrally or are subjected to purifying selection. *Actaea japonica*'s low genetic diversity probably results from a recent expansion, not a recent selective sweep on plastid genes.

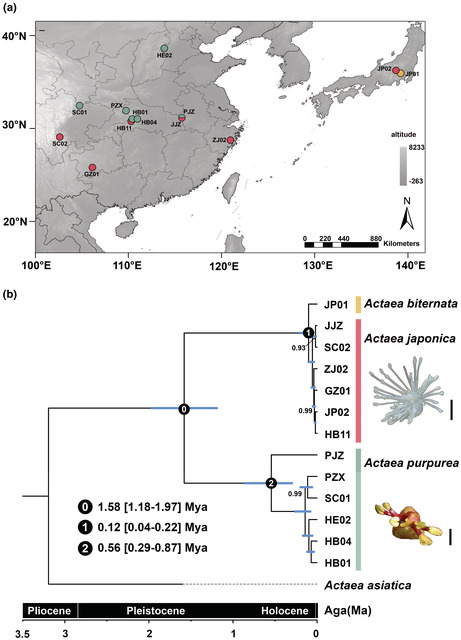

## 1 INTRODUCTION

The plant genomes are compartmentalized as other eukaryotes, composed of the nucleus and organelles. As the result of endosymbiosis, plastids and mitochondria have retained features of their ancestral genomes but also transferred most of their genes to the nuclear genome (Kleine et al., 2009). Genomes of plastids (plastomes) in photosynthetic angiosperms are relatively conserved in structure, gene number, and arrangement (Palmer & Stein, 1986). A typical angiosperm plastome is circular and could be split into four regions: a large single‐copy region (LSC) and a small single‐copy region (SSC), which are separated by two identical inverted repeats (IRs) (Davis et al., 2014; Du et al., 2015). It normally includes ca. 80 protein coding genes (PCGs), 30 transfer RNA genes and four ribosomal RNA genes (Daniell et al., 2016). PCGs encode proteins essential in photosynthesis, transcription, and translation (Kleine et al., 2009). Plastomes normally have two notable features: (1) there are many identical copies in plant cells, resulting in a high polyploidy; (2) virtually lack of recombination (Greiner et al., 2011), but there are still several genera present high recombination rates, which altered the plastome structure, e.g., *Onobrychis* Mill. and *Trifolium* L. (Cai et al., 2008; Moghaddam et al., 2022).

It is recognized that the sequence variation in plastomes is maintained by the mutation‐drift balance, and no adaptive evolution would have occurred (reviewed in Bock et al., 2014). However, recent evidence has shown that positive selection may have played an important role in the evolution of plastomes (Bock et al., 2014; De Santana Lopes et al., 2021; Wu et al., 2020). For example, Muir and Filatov (2007) inferred a selective sweep on the *Silene* L. plastome, occurring between 0.16 and 1.06 Mya (Million years ago). Sambatti et al. (2008) used reciprocal transplant experiments to show that plastid genes were involved in drought adaptation in *Helianthus petiolaris* Nutt. and *H. annuus* L. Several recent comparative genomic studies in a plethora of plant groups also detected signatures of positive selection from the patterns of sequence diversity (e.g., Ye et al., 2018; Zhao et al., 2020). Although these studies provided us with evidence for positive evolution of plastid genes at both the micro‐ and macro‐evolution levels, less studies have systematically investigated how plastomes have evolved during the speciation process. Plastomes could contribute to the speciation process through establishment of reproductive barriers by genetic incompatibility between the nucleus. For example, a recent study has shown that adaptation to specific environmental factors could cause the evolution of the hybridization barriers via the cytoplasm and nucleus incompatibility (Zupok et al., 2021).

*Actaea purpurea* (P.K. Hsiao) J. Compton is a perennial herb growing in the understory or the forest margins (Hsiao, 1979; Li & Brach, 2001). Its flowers are distinct from other species in the genus by having purple sepals and less stamens with purple filaments and yellow anthers, while flowers of other congeners are white and have numerous white stamens (Chang et al., 2020). Phylogenetic studies have shown that *A. purpurea* is sister to *A. japonica* Thunb. and *A. biternata* (Siebold & Zucc.) Prantl, and the three species formed a well‐supported clade (Compton, Culham, Gibbings et al., 1998, Compton, Culham, & Jury, 1998). Chang et al. (2020) used three plastid markers (*trnL‐trnF*, *rpl20‐rps12*, *trnS‐trnG*) to study genetic divergence of the group, and they found a striking pattern: all individuals of *A. japonica* and *A. biternata* shared one haplotype. Compared with *A. purpurea,* which had multiple haplotypes, the lack of genetic variation in *A. japonica* and *A. biternata* might be caused by a historical selective sweep, or a recent demographic expansion of *A. japonica* and *A. biternata* populations. If the selective sweep hypothesis is true, plastid genes may have played an important role in the divergence and speciation of *A. purpurea* and its relatives. To discriminate the two scenarios, we sequenced 13 plastomes of *A. purpurea* and its closest relatives, and conducted a systematic survey of positive selection in plastid genes of *A. japonica* + *A. biternata*, using both population genetic‐based test (the McDonald‐Kreitman test; McDonald & Kreitman, 1991) and codon‐based methods using maximum likelihood to estimate the ratio of nonsynonymous to synonymous substitutions (*ω*) across a phylogeny. We also investigated the structure and gene content variation in this group. Our work sheds light on the evolution of plastomes in divergence and speciation, and also on the evolutionary history of *A. purpurea* and its close relatives.

## 2 MATERIALS AND METHODS

### 2.1 Taxon sampling

Thirteen individuals representing 13 populations of the three species of *Actaea* L. were sampled in the study, covering all their distribution area (Table S1–S5). All voucher specimens were deposited in Shaanxi Normal University Herbarium (SANU). The latitude, longitude, and elevation of each sampling site were recorded using a hand‐held eTrex GPS (Garmin). Leaves from each individual were dried immediately in silica gel, and then stored at room temperature for further DNA extraction.

### 2.2 Sequencing, genome assembly, and gene annotation

According to the standard protocol provided by Illumina, the silica‐dried leaf material was sent to Novogene (Beijing, China) for library preparation and sequencing. In short, total genomic DNA was extracted from 20 to 30 mg silica‐gel dried leaves using a modified Cetrimonium bromide (CTAB) method (Doyle & Doyle, 1987). DNA samples were then subjected to ultrasonic treatment, mechanical cleavage, purification, end repair, adding adenylate to the 3′ end, and linker ligation to construct a sequencing library. Quality control of the library was executed by a Qubit 3.0 fluorometer (Life Technologies, Shanghai, China). NGS3K/Caliper and q‐PCR were also used to secure the quality of the sequencing library. Sequencing was performed using an Illumina‐Miseq Novaseq 6000. Double‐ended reads with a length of 150 bp were generated. The output data was subjected to data quality control by FastP (parameter: ‐q 30 ‐u 50) (Chen et al., 2018). The plastomes were de novo assembled utilizing GetOrganelle v1.7.5 (Jin et al., 2020), and the parameter was ‐R 30 ‐J 1 ‐M 1 ‐F embplant_pt. The obtained scaffold was visually corrected in the Bandage v0.8.1 (Wick et al., 2015) to obtain the complete plastome.

After genome assembly, we used Geneious R10 (Biomatters Ltd., Auckland, New Zealand) and CPGAVAS2 (Shi et al., 2019) to perform gene annotation. The plastome of *Actaea asiatica* Hara (Zhai et al., 2019) was used as the reference for gene annotation. We manually checked and modified the draft genome according to the reference genome and the result file of CPGAVAS2 to accurately define the boundaries between start and stop codons, as well as between gene exons and introns. tRNAscan‐SE v1.21 was used to verify annotated tRNA genes (Schattner et al., 2005). In order to visually show the structure and genomic content of plastomes of the three *Actaea* species, we made a circular illustration for each plastome using the Organellar Genome Draw program (OGRAW; https://chlorobox.mpimp‐golm.mpg.de/OGDraw.html) (Greiner et al., 2019).

### 2.3 Comparative analysis

We conducted multiple sequence alignment in MAFFT v7 (Katoh & Standley, 2013) with the default parameters (algorithm: Auto; scoring_matrix: 200PAM/*k* = 2; gap_open_penalty: 1.53; offset_value: 0.123), and subsequently checked manually in Geneious R10. In order to identify the interspecific structural variation between *A. purpurea* and its relatives, we used mVISTA (Frazer et al., 2004) to visualize the alignment. We chose the Shuffle‐Lagan mode in the setup and *A. biternata* as the reference. In addition, we used Mauve v2.3.1 (Darling et al., 2010) to confirm whether gene rearrangement events occurred among and within species. We also used IRscope (https://irscope.shinyapps.io/irapp/) (Amiryousefi et al., 2018) to detect the contraction and expansion of these boundaries. In order to distinguish differences in variation between different regions of the plastomes, DnaSP v6 (Rozas et al., 2017) was used to estimate the nucleotide diversity (*pi*) of all coding and noncoding regions (intergenic regions and introns).

### 2.4 Repetitive sequence analysis

Four types of repeats were searched for in the obtained plastomes: tandem repeats, dispersed repeats, palindrome repeats, and microsatellite sequences (SSR). Tandem Repeats Finder v4.09.1 (Benson, 1999) was used to search for tandem repeats with a length of at least 10 bp. The alignment parameters (match, mismatch, and indel) were set to 2, 7, and 7, respectively. We used REPuter software (Kurtz et al., 2001) to search for dispersed repeats and palindrome repeats, with the minimum repeat length of 30 bp, and the minimum interval between repeats of 3 bp. The minimum similarity between sequences was set to 90%. The MISA‐web (https://webblast.ipk‐gatersleben.de/misa/index.php?action=1) (Beier et al., 2017) was used to search for SSRs. The thresholds for single nucleotide, dinucleotide, trinucleotide, tetranucleotide, pentanucleotide, and dinucleotide were set to 10, 5, 4, 3, 3, and 3, respectively.

### 2.5 Phylogenetic and dating analysis

For the phylogenetic and dating analyses, we used the plastome of *A. asiatica* (Zhai et al., 2019) as the outgroup. Based on plastomes of all populations, maximum likelihood (ML) trees were constructed using IQtree v2.1.4 (Minh et al., 2020). The optimal nucleotide substitution model was determined by jModeltest v2.1 (Darriba et al., 2012) as GTR. Bootstrap values were assessed by ultrafast bootstrap approximation (UFBoot; Hoang et al., 2017) for 1000 replicates. We used an uncorrelated relaxed log‐normal molecular clock to estimate divergence times using BEAST v1.10 (Suchard et al., 2018). The program BEAUti was used to set the parameters for analysis. As there is no reliable fossil record for *A. purpurea* and its kin, we used a secondary‐calibration method. The separation of *A. purpurea* between *A. japonica* + *A. biternata* was set at 1.63 Mya (95% highest posterior density: 1.02–2.21 Mya; Chang et al., 2020). We run 100,000,000 generations of the chain, and sampled parameters every 1000 generations. The first 20% of the parameters were discarded as burn‐in. We then used Tracer v1.7.1 (Rambaut et al., 2018) to make sure the effective sampling size (ESS) for each parameter was larger than 200. Finally, Tree Annotator v1.7.1 (Suchard et al., 2018) was used to generate the maximum clade credibility (MCC) tree.

### 2.6 Detection of signatures of positive selection

Signatures of positive selection could be detected using several tests. A modest to high amount of sequence variation is often a prerequisite for most analyses. As our data set had limited sequence variation, we focused our tests on genes with enough variable sites. A combination of different tests would provide more reliable results. We first calculated Tajima's *D* (Tajima, 1989) and Fu's *F*_S_ (Fu, 1997) for each gene using the program ARLEQUIN v3.5.2.2 (Excoffier et al., 2005). The significance level was inferred with 1000 simulated samples. These tests cannot distinguish between selection and demographic dynamics, i.e., population bottlenecks or expansions, but significant values would indicate non‐neutral evolution of sequences detected.

The second method we used was the codon‐based method that estimates the ratio of nonsynonymous to synonymous (*ω*) across a phylogeny. We used EasyCodeML v1.0 (Gao, Chen, et al., 2019; Gao, Liu, et al., 2019) preset mode as the default setting. Then, we utilized the branch‐site model (Yang & Nielsen, 2002) to identify positively selected loci from genes in the foreground branch. The genes with *p* < .05 in the chi‐square test are selected as candidate positives. For both models, we used sequences of *A. japonica* and *A. biternata* as the foreground according to our hypothesis.

It is generally recognized that the codon‐based method is conservative, as adaptive sites would be diluted across the entire sequence. We thus used the McDonald‐Kreitman test (MKT) to complement the above analysis. This test calculates a neutrality index (NI) by dividing the ratio of nonsynonymous to synonymous polymorphisms within species to the ratio of nonsynonymous to synonymous divergence between species. A less than one value of NI would indicate positive selection. All MKTs were run using the MKT‐web (http://mkt.uab.es/mkt/MKT.asp) (Egea et al., 2008).

### 2.7 Environmental analysis

We used a total of 42 sampling sites based on our field collections, including 22 for *A. purpurea*, 18 for *A. japonica*, and one for *A. biternata* (Table S3) to conduct the environmental analysis. Nineteen contemporary environment variables (BIO1‐BIO19) were downloaded from the WorldClim website (http://worldclim.org/) (Hijmans et al., 2005). ArcGIS v10.5 was used to extract the values of 19 contemporary environmental variables and altitude for each sampling site. We then performed a Principal Component Analysis (PCA) using the R package FactoMineR (Lê et al., 2008), followed by plotting using the R package ggplot2 (Wickham, 2016). Data normality was checked by Shapiro–Wilk's test for each variable in R. For variables not normally distributed, we took the logarithm for them before the PCA. For the environmental variables that have a larger contribution to PCs, we used the Welch's t‐test and the Wilcoxon rank sum test in R to test whether the difference is significant between *A. purpurea* and *A. japonica* + *A. biternata*.

## 3 RESULTS

### 3.1 Characteristics of plastomes of *A. purpurea* and its relatives

**FIGURE 1:**
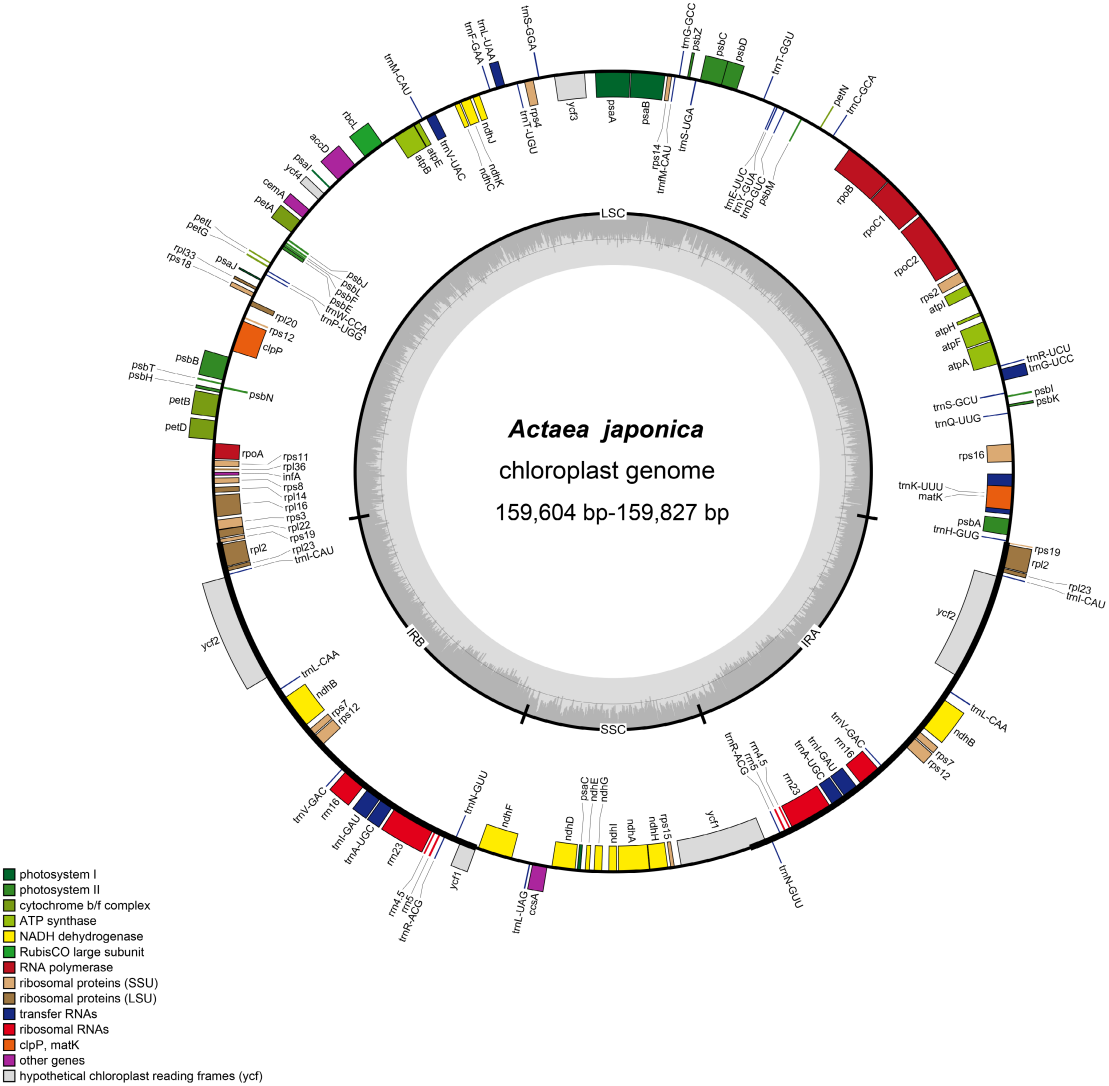
Gene map of the *Actaea japonica* plastome. Outside the circle are genes transcribed in a counter‐clockwise direction, whereas inside the circle are those transcribed in a clockwise direction. In the inner circle, the dark gray area represents GC content and the thick line indicates the extent of different regions. Different colors for genes show different functional groups. LSC, Large‐single‐copy; SSC, Small‐single‐copy; IR, Inverted repeat.

Thirteen complete plastomes of *A. purpurea* and its relatives were sequenced and annotated. These plastomes all possessed a typical angiosperm quadripartite structure (Figures 1, S1), including the LSC (88,586–88,984 bp), the SSC (17,490 bp‐17,763 bp), and two IRs (26,530‐26,652 bp). Among the 13 plastomes, population PJZ of *A. purpurea* (159,398 bp) had the smallest plastome, and HB11 of *A. japonica* (159,821 bp) had the largest (Table 1). The total GC content is 38.1%, and it was higher in the IR (43.1–43.0%) than both the LSC (36.2–36.3%) and SSC (32.3–32.6%) (Table 1).

**TABLE 1:** Characteristics of plastomes of *Actaea purpurea* and its relatives

Species	Population	Size(bp)				GC content%				Gene no.	PCG	tRNA	rRNA	Genes with introns	Pseudo‐gene no.
		Overall	LSC	SSC	IR	Overall	LSC	SSC	IR						
*Actaea biternata*	JP01	159,761	88,930	17,757	26,537	38.10%	36.20%	32.30%	43.10%	131	84 (5)	37 (7)	8 (4)	15	2
*A. japonica*	GZ01	159,735	88,900	17,762	26,536	38.10%	36.20%	32.30%	43.10%	131	84 (5)	37 (7)	8 (4)	15	2
	HB11	159,821	88,984	17,763	26,537	38.10%	36.20%	32.30%	43.10%	131	84 (5)	37 (7)	8 (4)	15	2
	JJZ	159,604	88,774	17,756	26,537	38.10%	36.30%	32.30%	43.10%	131	84 (5)	37 (7)	8 (4)	15	2
	JP02	159,786	88,955	17,757	26,537	38.10%	36.20%	32.30%	43.10%	131	84 (5)	37 (7)	8 (4)	15	2
	SC02	159,768	88,939	17,755	26,537	38.10%	36.20%	32.30%	43.10%	131	84 (5)	37 (7)	8 (4)	15	2
	ZJ02	159,725	88,894	17,757	26,537	38.10%	36.20%	32.30%	43.10%	131	84 (5)	37 (7)	8 (4)	15	2
*A. purpurea*	HB01	159,497	88,703	17,490	26,652	38.10%	36.30%	32.60%	43.00%	131	84 (5)	37 (7)	8 (4)	15	2
	HB04	159,385	88,586	17,495	26,652	38.10%	36.20%	32.60%	43.00%	131	84 (5)	37 (7)	8 (4)	15	2
	HE02	159,496	88,893	17,541	26,531	38.10%	36.20%	32.50%	43.10%	131	84 (5)	37 (7)	8 (4)	15	2
	PJZ	159,398	88,814	17,561	26,530	38.10%	36.30%	32.50%	43.10%	131	84 (5)	37 (7)	8 (4)	15	2
	PZX	159,435	88,793	17,543	26,531	38.10%	36.30%	32.50%	43.10%	131	84 (5)	37 (7)	8 (4)	15	2
	SC01	159,574	88,780	17,490	26,652	38.10%	36.30%	32.60%	43.00%	131	84 (5)	37 (7)	8 (4)	15	2

*Note*: Numbers in brackets mean no. of duplicated genes.

Abbreviation: PCG, protein coding genes.

The number of genes in different *Actaea* species was also consistent: each plastome comprised 131 predicted genes, 18 of which were repeated in IRs. The 113 unique genes included 79 PCGs, 30 tRNA genes, and four rRNA genes. The incompletely duplicated copies of *ycf1* and *rps19* in IR were two pseudogenes (Table 1; Figures 1, S1–S7). A total of 12 genes (excluding three duplicate copies) contained introns, of which nine genes had intron (*atpF*, *ndhA, ndhB, petB*, *petD*, *rpl2, rpl16, rpoC1*, *rps16*) and three genes had two introns (*ycf3*, *clpP*, *rps12*) (Table S2).

**FIGURE 2:**
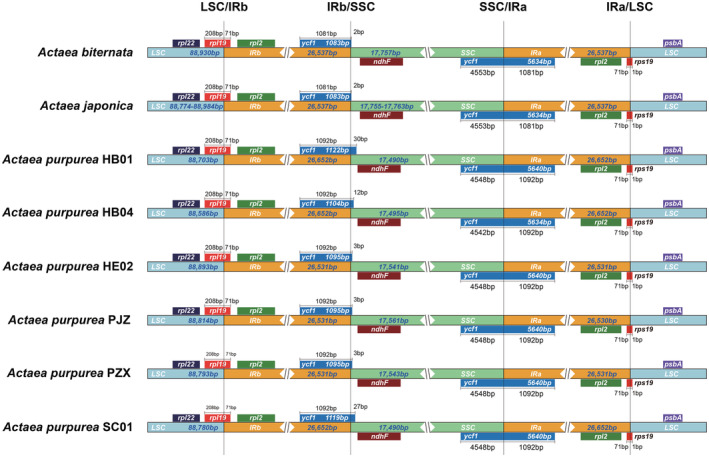
Gene locations at region boundaries in plastomes of *Actaea purpurea* and its relatives.

The comparison of border regions of *Actaea* plastomes showed that IRs were relatively stable, and there was no significant expansion or contraction events. The LSC‐IRb and IRa‐LSC boundaries were located in two copies of the *rps19* gene, respectively, and no displacement was detected. IRb‐SSC and SSC‐IRa boundaries were located in two copies of the *ycf1* gene (Figure 2). The exact location of the boundaries of all populations was constant in *A. japonica* and *A. biternata*. However, in *A. purpurea* populations, the IRb‐SSC and the IRa‐LSC boundary had shifted to varying degrees (Figure 2).

### 3.2 Structural and sequence diversity of *Actaea* plastomes

**FIGURE 3:**
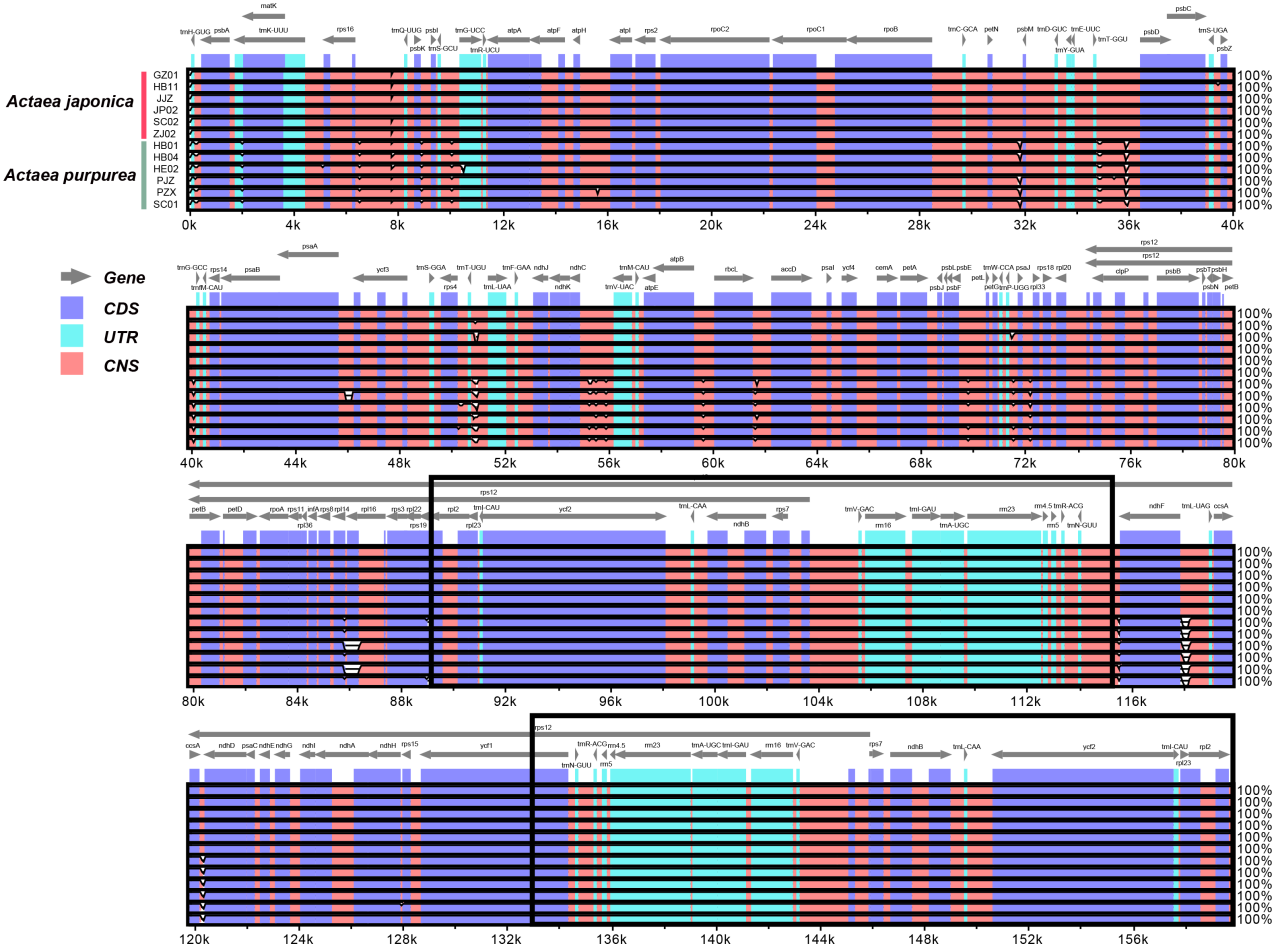
Visualization of the alignment of *Actaea* plastomes by mVISTA. *Actaea biternata* was set as the reference. The gray arrows above represent genes. Different colors represent different regions (coding and noncoding). Position in the genome is shown on the horizontal axis at the bottom of each block. Alignment similarity percentages are shown on the right side of the graph (the vertical axis). Two black frames indicate the two IR regions.

Taking *A. biternata* as the reference, the results of mVISTA showed that all *Actaea* plastomes have high sequence similarity. Most of the differences existed between the inter‐specific divergence of *A. japonica* and *A. purpurea*, while the intraspecific difference was very small. Most of these differences were located in the noncoding regions, and the region with the highest *pi* value was in the noncoding region: *rpl14*‐*rpl16* (Figure 3). Mauve's multiple comparative analysis of 13 chloroplast genomes showed that no genome rearrangement event had occurred (Figure S2).

**FIGURE 4:**
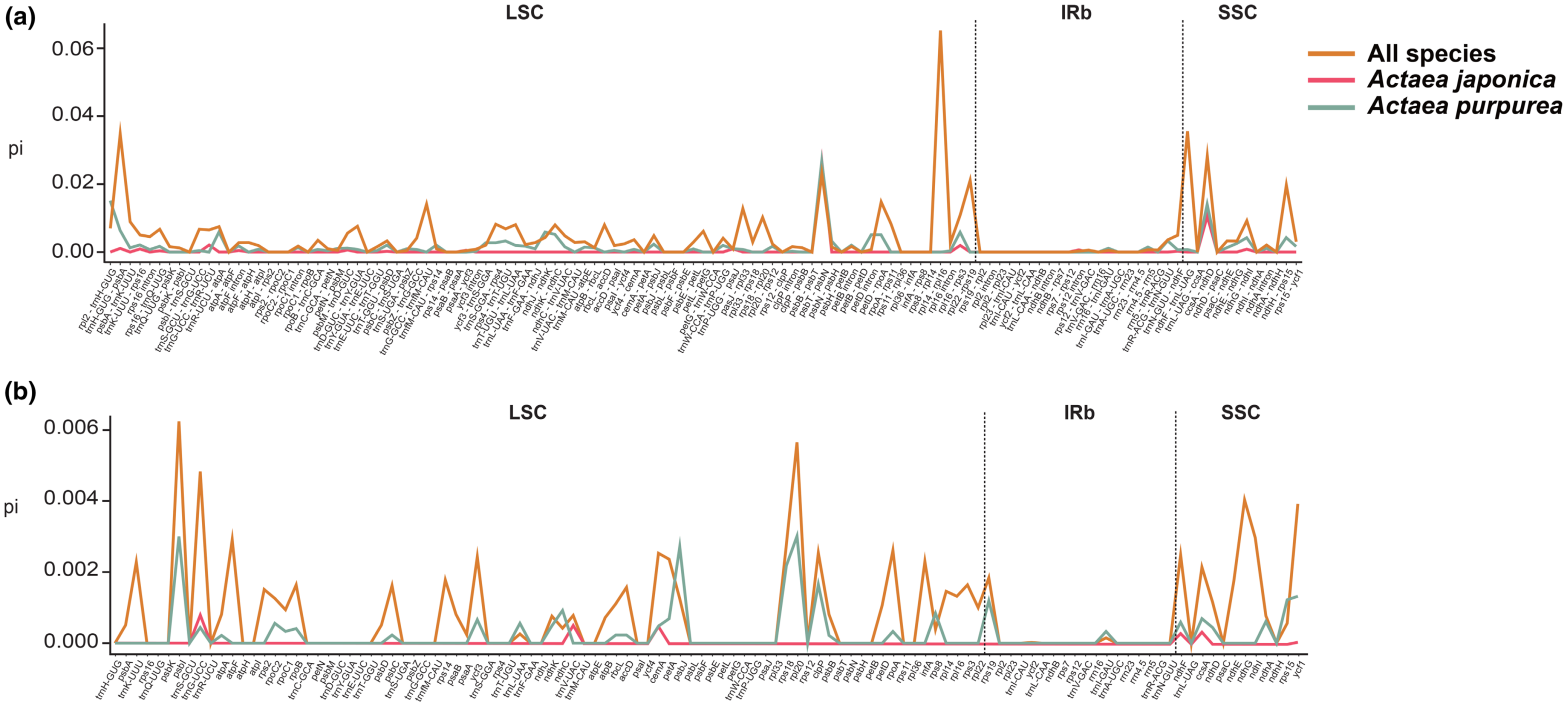
Comparison of nucleotide polymorphisms across *Actaea* plastomes. (a) Coding regions; (b) noncoding regions, i.e., intergenic regions and introns

The five genes with the highest sequence diversity were *psbI*, *rpl20*, *trnG* (UCC), *ndhG*, and *ycf1*. The corresponding noncoding regions were *rpl14*‐*rpl16*, *ndhF*‐*trnL*(UAG), *trnH*(GUG)‐*psbA*, *ccsA*‐*ndhD*, and *psbT*‐*psbN* (Figure 4). Both the noncoding and coding regions of *A. japonica* had significant lower genetic diversity (0.00040 and 0.00002) than *A. purpurea* (0.001425, *p* = 4.8E‐11; 0.00023, *p* = 9.1E‐06) (Table 2). In addition, consistent with the comparison based on the complete plastome (Figure 3), the nucleotide diversity of the noncoding region (0–0.06527, 0.04381) was significantly higher than the nucleotide diversity of the coding region (0–0.00624, 0.00078, *p* = 3.7E‐7) (Figure 4). Meanwhile, the nucleotide diversity of the IR region (noncoding region: 0.00089; coding region: 0.00001) was lower than that of the LSC (0.00471, *p* = 5.2E‐5; 0.00081, *p* = 3.1E‐3) and the SSC (0.00871, *p* = .012; 0.00165, *p* = 2.1E‐4) (Table 2). At the whole plastome level, the nucleotide diversity of *A. purpurea* is higher than that of *A. japonica* (Table 2).

**TABLE 2:** Nucleotide diversity (*pi*) across the 13 plastomes from *Actaea purpurea* and its relatives

		LSC	SSC	IR	LSC + SSC + IR
Noncoding region	All species	0.00471 (0–0.06527)	0.00871 (0–0.03559)	0.00842 (0–0.00496)	0.00438 (0–0.06527)
	*Actaea japonica*	0.00041 (0–0.02667)	0.00095 (0–0.01054)	0.00089 (0–0.00062)	0.00040 (0–0.02667)
	*Actaea purpurea*	0.00154 (0–0.02667)	0.00249 (0–0.01406)	0.00235 (0–0.00332)	0.00142 (0–0.02667)
Coding region	All species	0.00081 (0–0.00624)	0.00165 (0–0.00403)	0.00001 (0–0.00015)	0.00078 (0–0.00624)
	*Actaea japonica*	0.00002 (0–0.00079)	0.00006 (0–0.00034)	0.00000	0.00002 (0–0.00079)
	*Actaea purpurea*	0.00026 (0–0.00301)	0.00041 (0–0.00132)	0.00002 (0–0.00033)	0.00024 (0–0.00301)
All regions	All species	0.00227	0.00361	0.00022	0.00211
	*Actaea japonica*	0.00010	0.00026	0.00001	0.00010
	*Actaea purpurea*	0.00069	0.00118	0.00014	0.00065

### 3.3 Repetitive sequences

**FIGURE 5:**
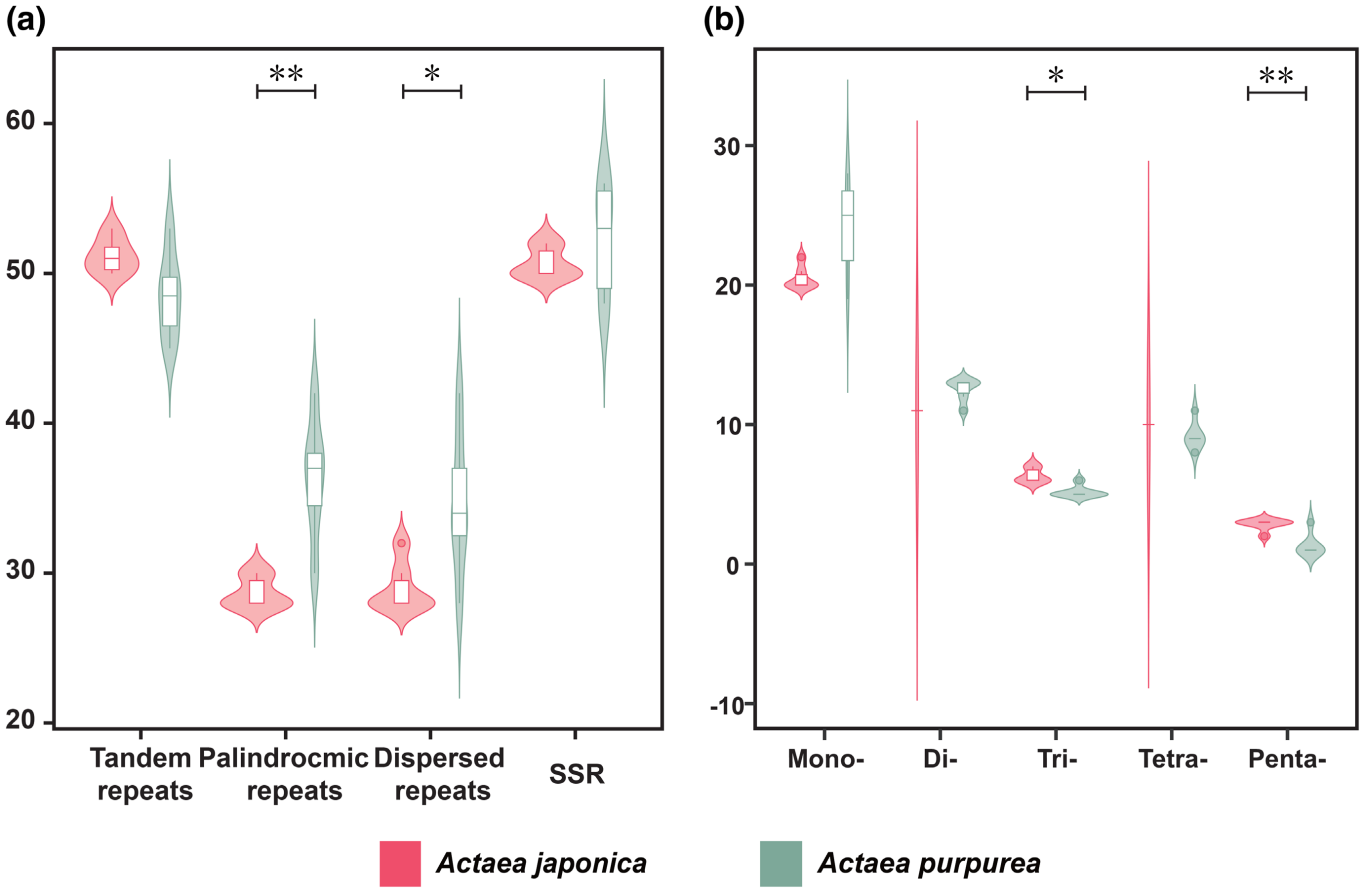
The number of tandem, palindromic, dispersed repeats and SSR in plastomes of *Actaea purpurea* and its relatives. (a) The number of four types of repeats; (b) the number of different SSR types.

The distribution of tandem repeats, dispersed, palindromic repeats, and SSR sequences in three *Actaea* species was analyzed. The repetitive sequences were mainly distributed in the LSC, and others were in the SSC and IRs. The number of dispersed repeats and palindromic repeats of *A. purpurea* was significantly higher than that of *A. japonica*, and the difference mainly existed in the SSC (Figures 5, S3). Except for SSRs, there was a significant gap between the three repeats of *A. purpurea* and *A. japonica* in the CDS region (Figure S3). We found 624 SSRs, including mononucleotide, dinucleotide, trinucleotide, tetranucleotide, and pentanucleotide. The numbers of each type were 289, 152, 76, 125, and 28, respectively. Mononucleotide accounted for 43.13% of all SSRs. *A. purpurea* had a unique tetranucleotide type and a unique pentanucleotide type (Figure S4). In addition, there was a 242 bp long repetitive sequence in *A. purpurea* (Figure S5).

### 3.4 Phylogeny and dating analysis

**FIGURE 6:**
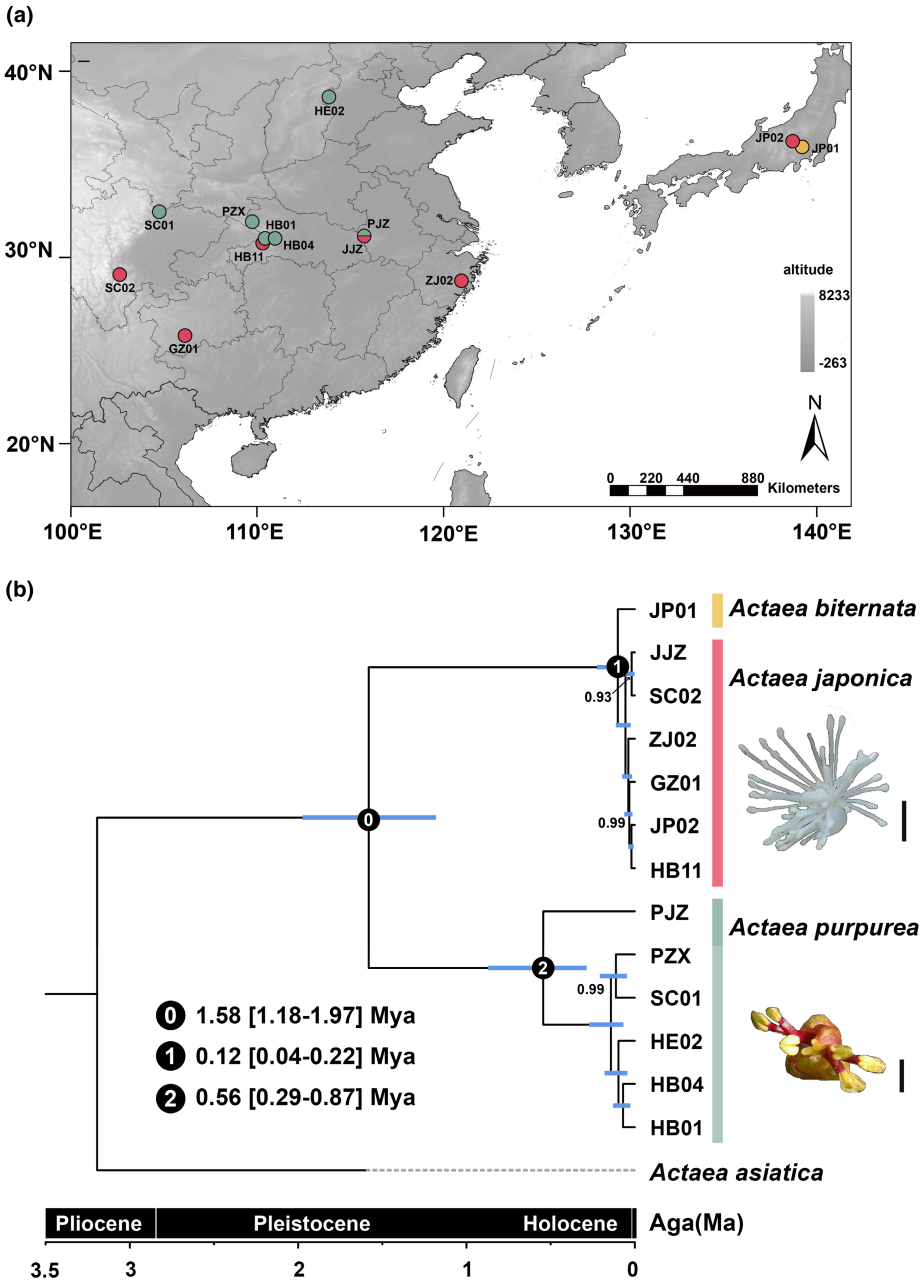
Sample distribution and divergence time of *Actaea purpurea* and its relatives based on the plastome data. (a) Sampling sites of the 13 populations; (b) divergence time estimation. Blue bars indicate the 95% highest posterior density intervals. The number on each node represents the posterior probability, and only values less than 1 are shown. Scale bar = 5 mm.

The time tree constructed based on the complete chloroplast genome showed that *A. purpurea* diverged from *A. japonica* and *A. biternata* at ca. 1.58 Mya (95% HPD: 1.18–1.97 Mya) and the latter two formed a clade (Figures 6b, S6). The divergence of *A. japonica* and *A. biternata* was at ca. 0.12 Mya (95% HPD: 0.04–0.23 Mya; node 1, Figure 6b), which was at the late Pleistocene. PJZ was the first *A. purpurea* population to separate from other populations about 0.56 Mya (95% highest posterior density: 0.29–0.87 Mya; node 2, Figure 6b).

### 3.5 Positive selection analysis

As positive selection tests require a modest to high amount of sequence variation between taxa, we only reported test results in 12 genes with suitable number of variable sites. Our results showed that no Tajima's *D* or Fu's *F*s values were significantly positive or negative (Table 3). Branch sites tests indicated that most plastid genes have evolved in a neutral (*ω* = 1) way or under purifying selection (*ω* < 1). Two genes (*rpoC1* and *rpoC2*) had a global *ω* > 1, but the two‐ratio model was not significantly better than model 0 (Table 3). Insufficient variation in our sequence data resulted in infinite or undefined NI values in MKTs. Other tests yielded nonsignificant values of NI (Table 3). Notably, the *rbc*L gene had a NI < 1 with a marginal significant level (*p* = .08). In summary, our positive selection analysis showed no definite evidence for adaptive evolution of plastid genes.

**TABLE 3:** Genetic variation in selected plastid genes of *Actaea purpurea* and its relatives and results from molecular tests of selection.

Gene	MKTs		Neutrality tests		Branch site model		
	NI	*p*‐value	Tajima's *D*	Fu's *F*s	*ω*_2_	Model A ln L	Model A null ln L
*matK*	0.14	.23	1.40	3.76	na	−2041.68	−2041.68
*ndhA*	na	.39	−0.39	0.44	na	−1441.96	−1441.96
*ndhD*	**na**	**.05**	1.20	0.73	na	−2022.81	−2022.81
*ndhF*	0.80	.85	0.97	0.55	na	−3010.97	−3011.19
*petA*	**na**	**.02**	1.48	1.40	na	−1322.47	−1322.47
*rbcL*	0.00	.08	2.04	1.83	na	−1923.33	−1923.33
*rpl20*	1.01	1.00	1.87	2.45	na	−492.67	−492.67
*rpoB*	7.04	.16	1.49	4.17	na	−4353.75	−4353.74
*rpoC1*	na	.36	0.69	0.97	na	−2746.05	−2746.05
*rpoC2*	0.33	.42	0.64	1.40	na	−5618.74	−5618.74
*ycf1*	2.87	.21	1.66	2.33	na	−7624.84	−7625.17
*ycf3*	na	.39	0.86	1.22	na	−691.21	−691.21

*Note*: Significant values are in bold.

Abbreviations: MKTs, McDonald‐Kreitman tests; NI, neutrality index; na, the data was infinite or undefined.

### 3.6 Environmental analysis

In the PCA of the 20 environments, PC1 and PC2 explained 77.01% of the total variation (Figure S7). *Actaea purpurea* and *A. japonica* + *A. biternata* were divergent in PC1 but not in PC2 (Figure S7). The main contribution of PC1 (58.62% variation) was from environmental variables related to both precipitation and temperature, with BIO12, BIO14, and BIO16 as the most important precipitation factors, and BIO9, BIO6, and BIO11 as the main temperature variables (Table S4). All these bioclimatic variables were significantly different between *A. purpurea* and *A. japonica* + *A. biternata* (Table S5). PC2 (18.39% of the variation) mainly captured the remaining temperature‐related environmental variables (BIO5, BIO8, and BIO10) and altitude (Table S4). These variables were not significantly different between the two groups, except BIO10 (Table S5).

## 4 DISCUSSION

### 4.1 Genome size, gene order and number were highly conserved among *A. purpurea* and its relatives

Plastomes are normally conserved in genome size, gene order, and number, especially in close‐related species. The plastomes of the three *Actaea* species all show a typical quadripartite structure, and belong to type I of Zhai et al. (2019). Also consistent with the previous study, a total of 131 genes were annotated, with 84 PCGs, 30 tRNA genes, 4 rRNA genes, and two pseudogenes. No inversions were detected among species either. These results demonstrate the conservation nature of plastomes between sister species. The overall GC contents of these plastomes are similar to those of other angiosperms (Palmer, 1991; Wolf et al., 2011), with the IR region possessing a higher GC content. This is because rRNA genes, which have high GC content, are located in the IR region (Raman et al., 2017).

IR/SSC and IR/LSC boundary shifts between species are common in plethora plant groups (e.g., Ye et al., 2018; Zhao et al., 2020). Length variation of the IR region is responsible for these differences, which is very crucial in stabilizing the plastome structure (Maréchal & Brisson, 2010). However, in some Leguminosae species such as *Trifolium subterraneum* L., the IR region was even completely lost (Cai et al., 2008; Lavin et al., 1990; Palmer et al., 1987). There were no boundary shifts within *A. japonica* and *A. biternata*, consistent with their low sequence diversity, while in *A. purpurea*, SSC‐IRb border shifts of 3–30 bp were detected. Between species, clear shifts of boundaries were detected at both borders, indicating that the *A. purpurea* and its relatives are indeed genetically divergent.

Gene loss or pseudogenization is common in plasto of seed plants (Jansen & Ruhlman, 2012). For example, among the six genes lost or pseudogenized in Ranunculaceae, *rpl32* and *rps16* were lost or pseudogenization multiple times in gymnosperms and angiosperms; *accD* and *infA* were also lost or pseudogenization in monocotyledon and eudicotyledon, while *rps7* was lost in *Passiflora* L. (Jansen & Ruhlman, 2012). Only the *trnT‐UGU* gene has been found to be lost in Trib. Anemoneae of Ranunculaceae (Zhai et al., 2019). In our study, the two pseudogenes, *ycf1* and *rps19*, were located in the IRb/SSC and IRa/LSC boundary regions, respectively. The formation of these two pseudogenes may be related to the change in the length of the IR region.

As revealed by the previous study (Chang et al., 2020), genetic diversity (measured by *pi* in this study) of *A. japonica* plastomes was much lower than that of *A. purpurea* (Figure 3), both in coding and noncoding regions. In both species, IRs were more conserved and exhibited lower genetic diversity than the LSC and SSC as previously reported (Ye et al., 2018; Zhao et al., 2020). The mechanism accounted for the slower substitution rate of IRs may be copy correction between IRs and the purging of deleterious mutation by gene conversion (Khakhlova & Bock, 2006). Predicted by the neutral theory of molecular evolution, noncoding regions of plastomes would have higher substitution rate. This is also true in our data set. The five most divergent noncoding regions were *rpl14‐rpl16*, *ndhF‐trnL* (UAG), *trnH*(GUG)‐*psbA*, *ccsA*‐*ndhD*, and *psbT*‐*psbN*. Among these regions, *trnH*‐*psbA* has been a classic plastid marker in many phylogenetic and phylogeographic studies (Shaw et al., 2014). Except that, the remaining four regions were not listed as the most variable regions across angiosperm lineages (Shaw et al., 2014). This shows that there may not a universally hyper variable region that can be used across all angiosperm groups. Instead, which region accumulated more substitutions might be lineage specific. The newly identified noncoding regions as well as SSR markers could be used to study phylogenetic relationships and population genetics within the genus *Actaea*.

### 4.2 Nonadaptive molecular evolution of plastome during speciation

We found that during the divergence and speciation of *A. purpurea* and *A. japonica* + *A. biternata*, the genetic variation of plastomes was indeed maintained by the mutation‐drift balance. Most molecular evolution tests failed to reject neutral evolution of the plastome in the divergence of *A. purpurea* and its relatives. Functional genes related to photosynthesis, translation, and other functions are all under strong purifying selection. This is in contrast to other recent studies that identified a few positively selected genes, e.g., *rbc*L (Lee‐Yaw et al., 2019; Liu et al., 2012) and *ndh* genes (Zhao et al., 2020). Environmental analysis indicated that *A. purpurea* and *A. japonica* were significantly divergent in most precipitation and temperature‐related variables, but we did not detect any adaptive signals in the plastomes of *A. japonica* + *A. biternata*. It is likely that the adaptive divergence of *A. purpurea* and *A. japonica* + *A. biternata* was mainly driven by the nuclear genome. Thus, whether plastid genes contribute to the divergence of populations and species seems lineage‐dependent. Nonadaptive evolution of plastid genes in our system also indicate that cytoplasmic incompatibility may not be the main mechanism of reproductive isolation between *A. purpurea* and its relatives.

In our data set, no fixed differences in transfer RNAs or ribosomal DNAs between species were detected, which is not unexpected giving these genes conserved function. The population genetic‐based and dN/dS‐based methods yielded inconsistent results. No positively selected genes were detected in the dN/dS‐based method, as in all cases, the alternative models were not significantly better than the null models. Insufficient variation in our sequence data resulted in infinite or undefined NI values in MKTs. There were four genes with NI < 1 in MKTs, but none of them were significant. We note that the *rbcL* gene had an NI < 1 with a marginal significant level (*p* = .08). Thus, it is possible that this gene may participate in the adaptation to different conditions for photosynthesis between *A. purpurea* and its relatives, considering their large distribution range.

*Actaea purpurea* is distributed in the northern part of the group's distribution, while *A. japonica* is in the south, and *A. biternata* only in Japan (Figure 6a). The distribution pattern is largely parapatric: there appears to be a barrier corresponding to the Sichuan Basin, the Yangtze River, and the Dabie Mountains separating the two groups, while both *A. purpurea* and *A. japonica* can be found in Hubei and Anhui Provinces (Chang et al., 2020). If the lack of genetic variation in plastid markers of *A. japonica* + *A. biternata* (all with the same haplotype) is not caused by a historical selective sweep, then a recent demographic expansion would be the only explanation. This means that the current parapatric distribution was formed by expansion of *A. japonica* + *A. biternata* from a possible previously sympatric population. In other words, the initial phase of divergence between *A. purpurea* and *A. japonica* + *A. biternata* most probably occurred in sympatry. This is consistent with the results of demographic modeling, showing that there has been continuous gene flow after the divergence of the two species (Chang et al., 2020). The expansion of *A. japonica* and *A. biternata* populations might be very recent, as the time for drift or selection to accumulate substitutions was limited. It probably occurred in the Pleistocene, when the climatic oscillations often drove changes in geographic distribution changes of many plant species (Hewitt, 2000, 2004; Qiu et al., 2011).

## AUTHOR CONTRIBUTIONS

**Danqing Zhang:** Data curation (lead); formal analysis (lead); investigation (lead); writing – original draft (equal). **Yi Ren:** Conceptualization (supporting); supervision (equal). **Jian‐Qiang Zhang:** Conceptualization (lead); funding acquisition (lead); writing – original draft (equal).

## CONFLICT OF INTEREST

All authors claim no conflict of interest.

## Supporting information


Figure S1–S7

Table S1–S5

## Data Availability

DNA sequences: newly generated plastomes were deposited in the GenBank database with accession numbers OM460061–OM460073.
