# Supplementary material for: Nonadaptive molecular evolution of plastome during the speciation of Actaea purpurea and its relatives
Source: Ecol Evol. 2022 Sep 17;12(9):e9321. doi: 10.1002/ece3.9321 (PMC9482002; doi:10.1002/ece3.9321)
Supplement: Supplementary file 1 — Figure S1–S7 [file ECE3-12-e9321-s001.pdf]

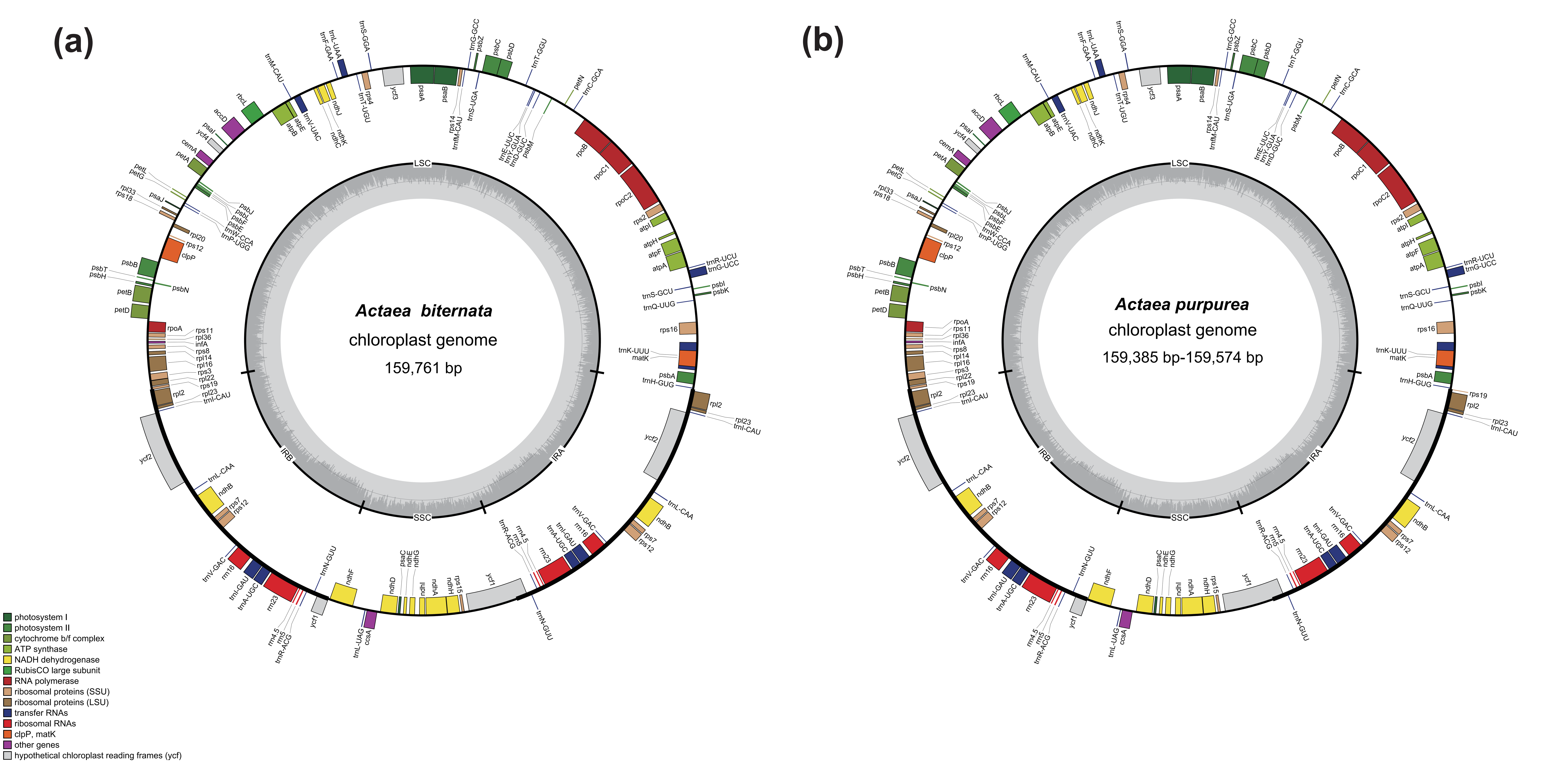
(a)
Actaea biternata
chloroplast genome
159,761 bp
LSC
SSC
IRA
IRB
(b)
Actaea purpurea
chloroplast genome
159,385 bp-159,574 bp
LSC
SSC
IRA
IRB
photosystem I
photosystem II
cytochrome b/f complex
ATP synthase
NADH dehydrogenase
RubisCO large subunit
RNA polymerase
ribosomal proteins (SSU)
ribosomal proteins (LSU)
transfer RNAs
ribosomal RNAs
clpP, matK
other genes
hypothetical chloroplast reading frames (ycf)

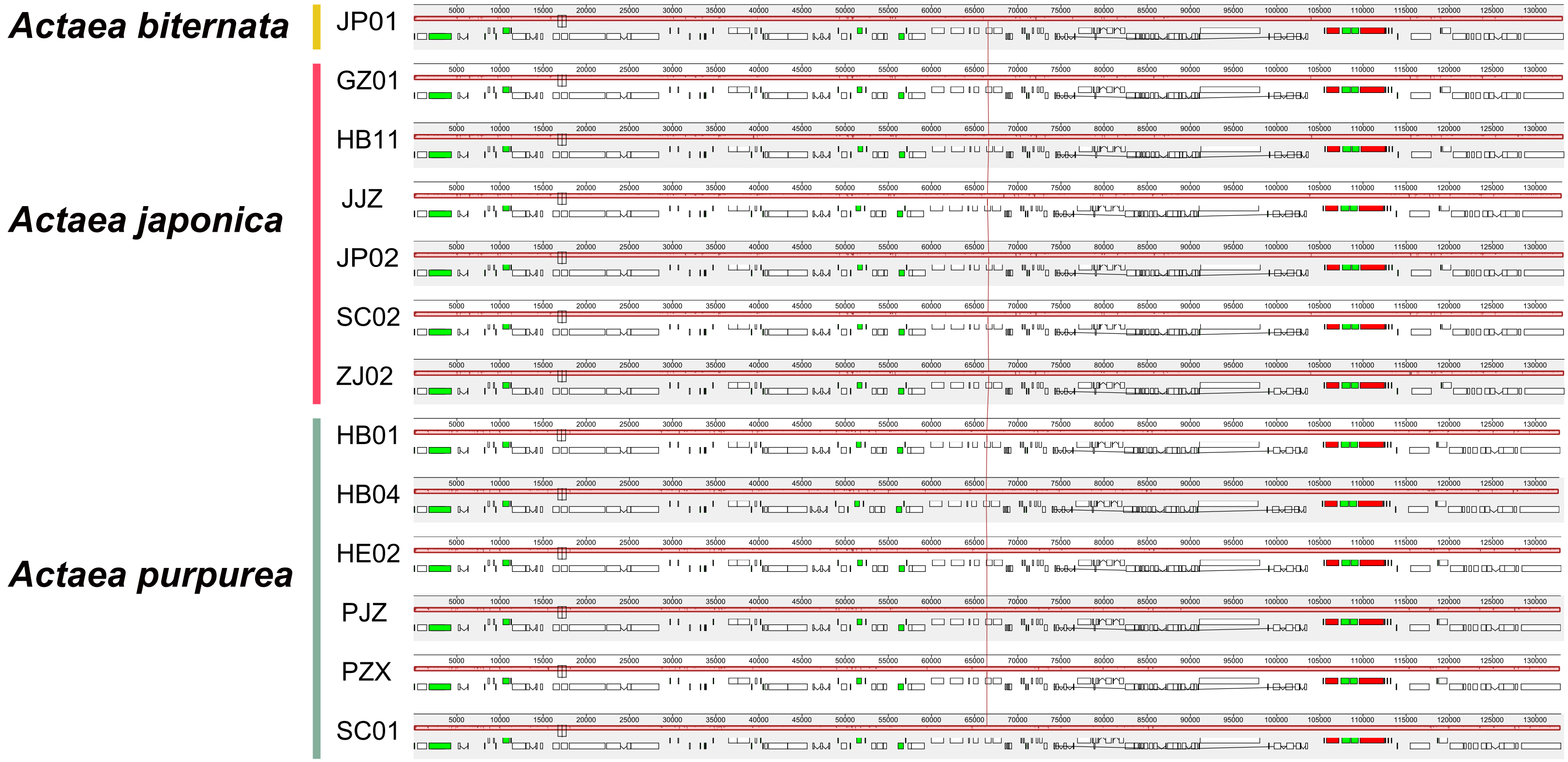

Actaea biternata
JP01
Actaea japonica
GZ01
HB11
JJZ
JP02
SC02
ZJ02
Actaea purpurea
HB01
HB04
HE02
PJZ
PZX
SC01

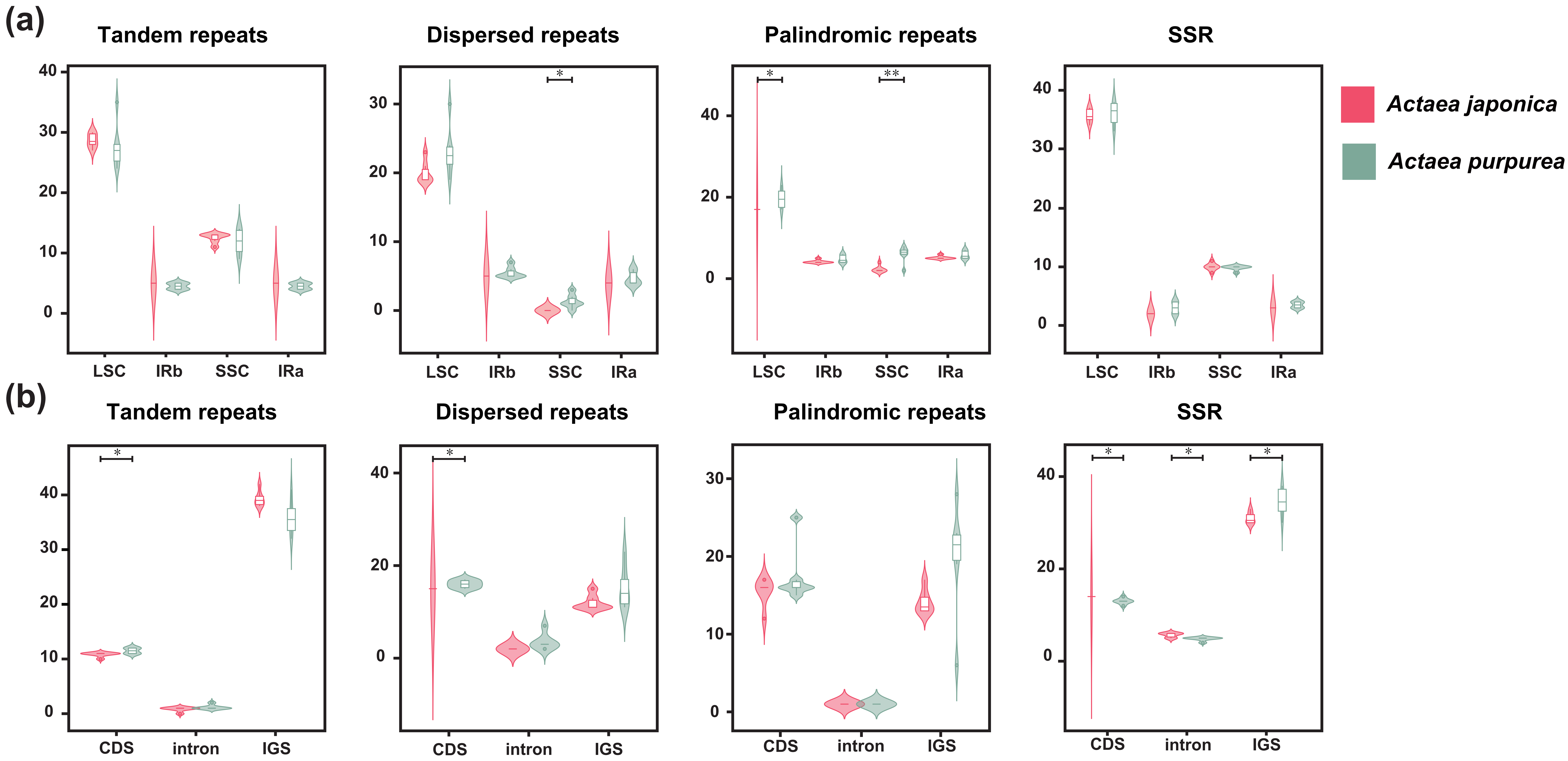

(a)
Tandem repeats
Dispersed repeats
Palindromic repeats
SSR
LSC
IRb
SSC
IRa
Actaea japonica
Actaea purpurea
(b)
Tandem repeats
Dispersed repeats
Palindromic repeats
SSR
CDS
intron
IGS

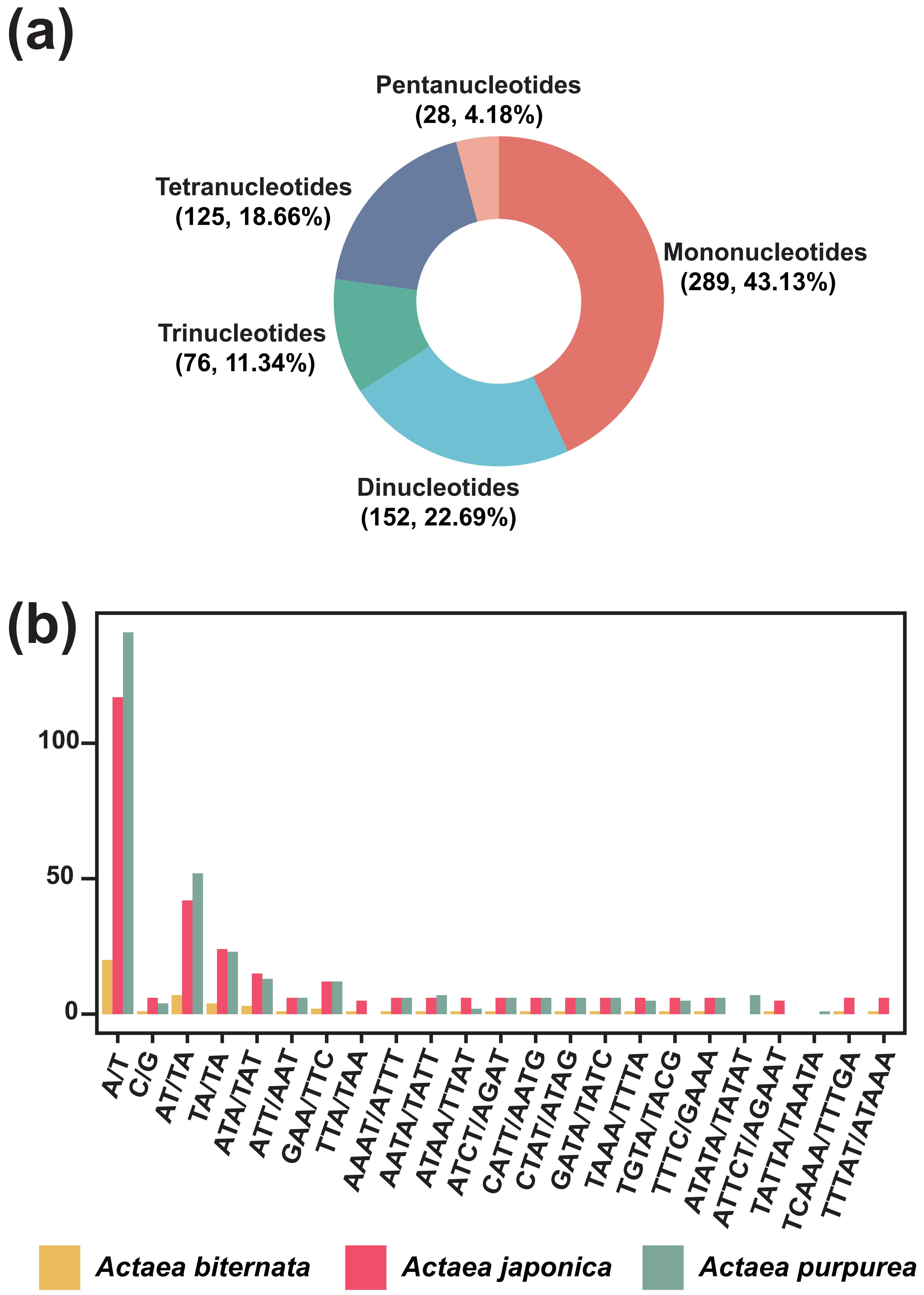
(a)
Pentanucleotides
(28, 4.18%)
Tetranucleotides
(125, 18.66%)
Mononucleotides
(289, 43.13%)
Trinucleotides
(76, 11.34%)
Dinucleotides
(152, 22.69%)
(b)
100
50
0
A/T
C/G
AT/TA
TA/TA
ATA/TAT
ATT/AAT
GAA/TTC
TTA/TAA
AAAT/ATTT
AATA/TATT
ATAA/TTAT
ATCT/AGAT
CATT/AATG
CTAT/ATAG
GATA/TATC
TAAA/TTTA
TGTA/TACG
TTTC/GAAA
ATATA/TATAT
ATTCT/AGAAT
TATTA/TAATA
TCAAA/TTTGA
TTTAT/ATAAA
Actaea biternata
Actaea japonica
Actaea purpurea

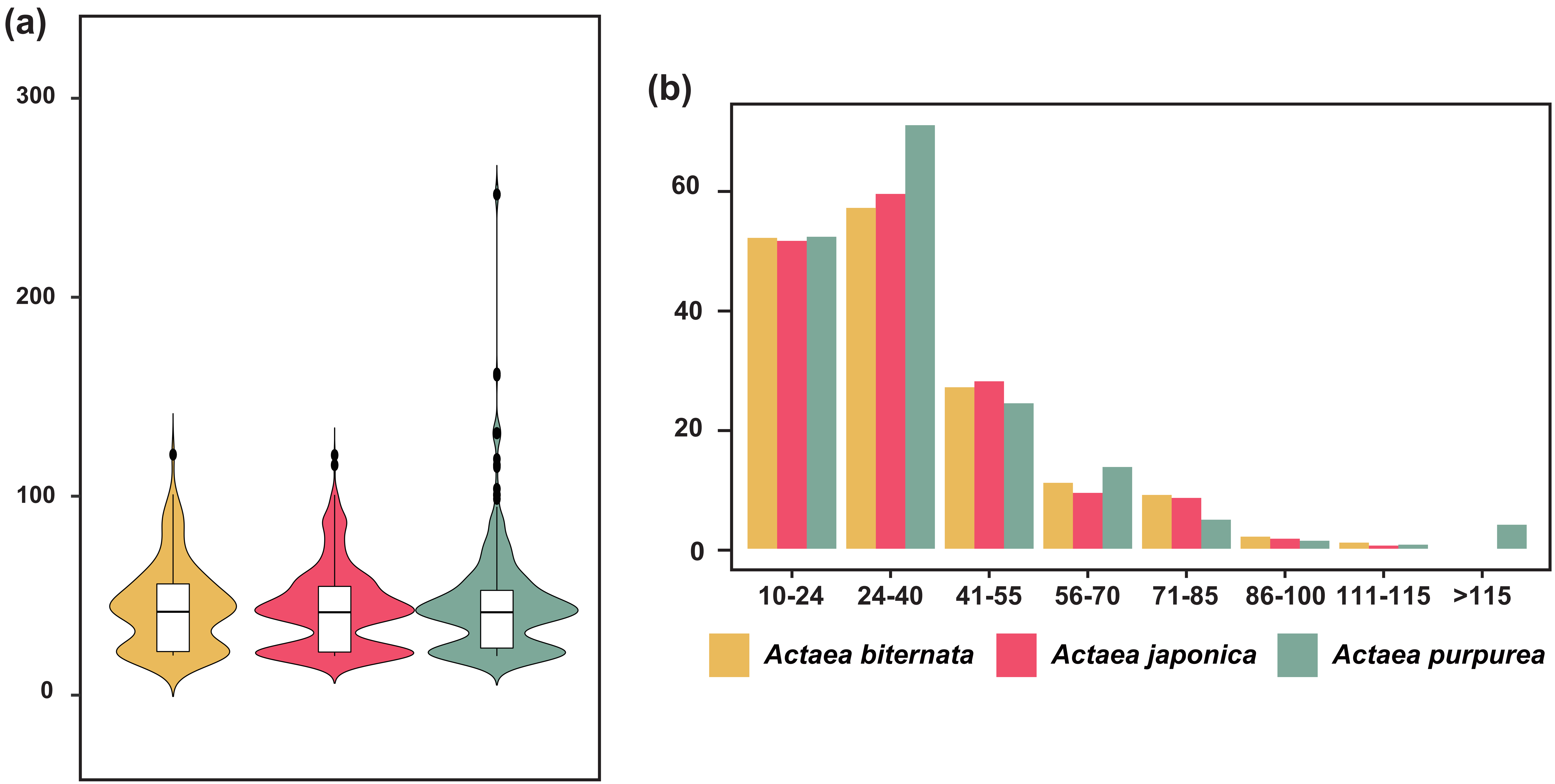

(a)
300
200
100
0
(b)
60
40
20
0
10-24
24-40
41-55
56-70
71-85
86-100
111-115
>115
Actaea biternata
Actaea japonica
Actaea purpurea

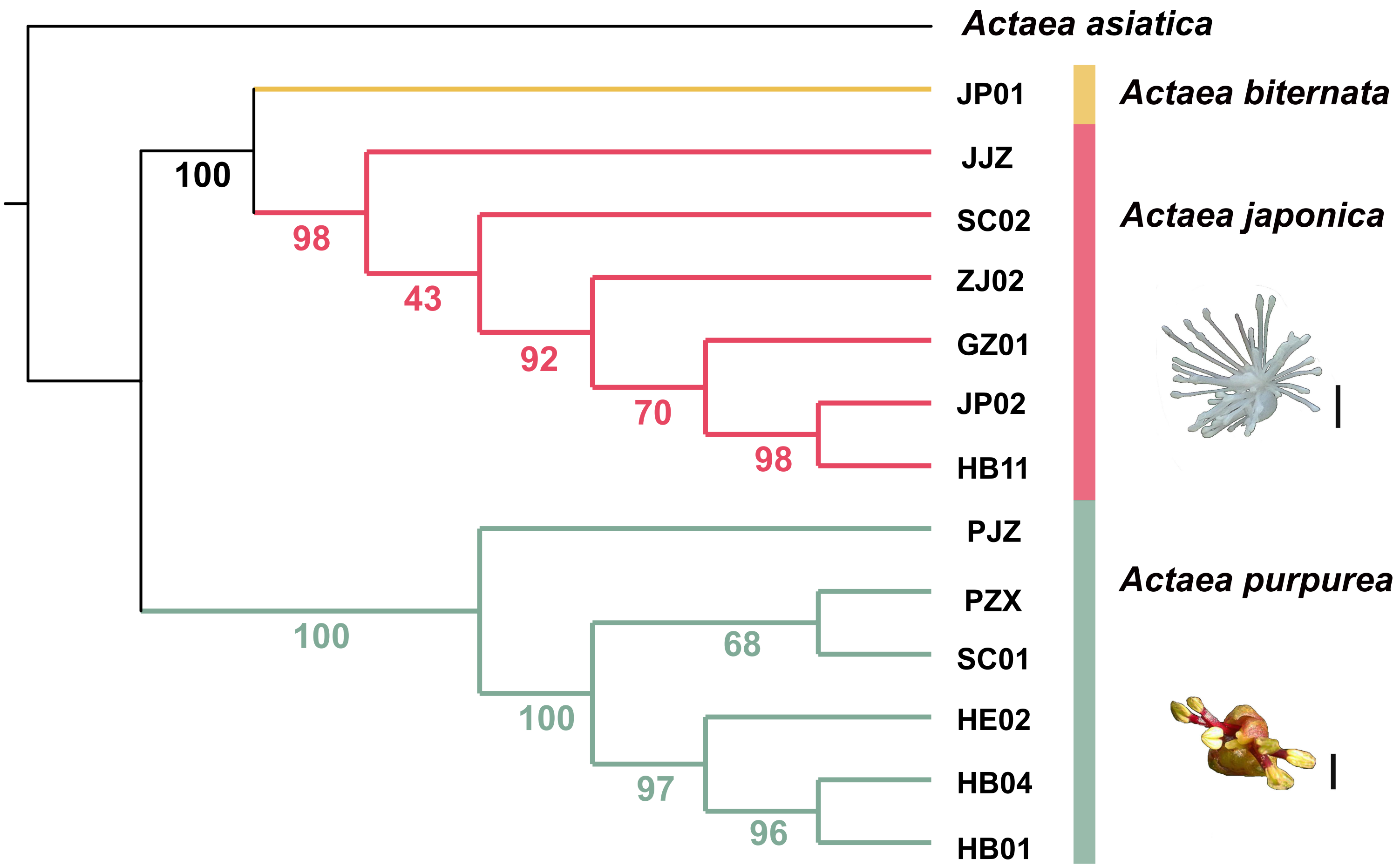
Actaea asiatica
JP01
JJZ
SC02
ZJ02
GZ01
JP02
HB11
PJZ
PZX
SC01
HE02
HB04
HB01
Actaea biternata
Actaea japonica
Actaea purpurea
100
98
43
92
70
98
100
100
68
97
96

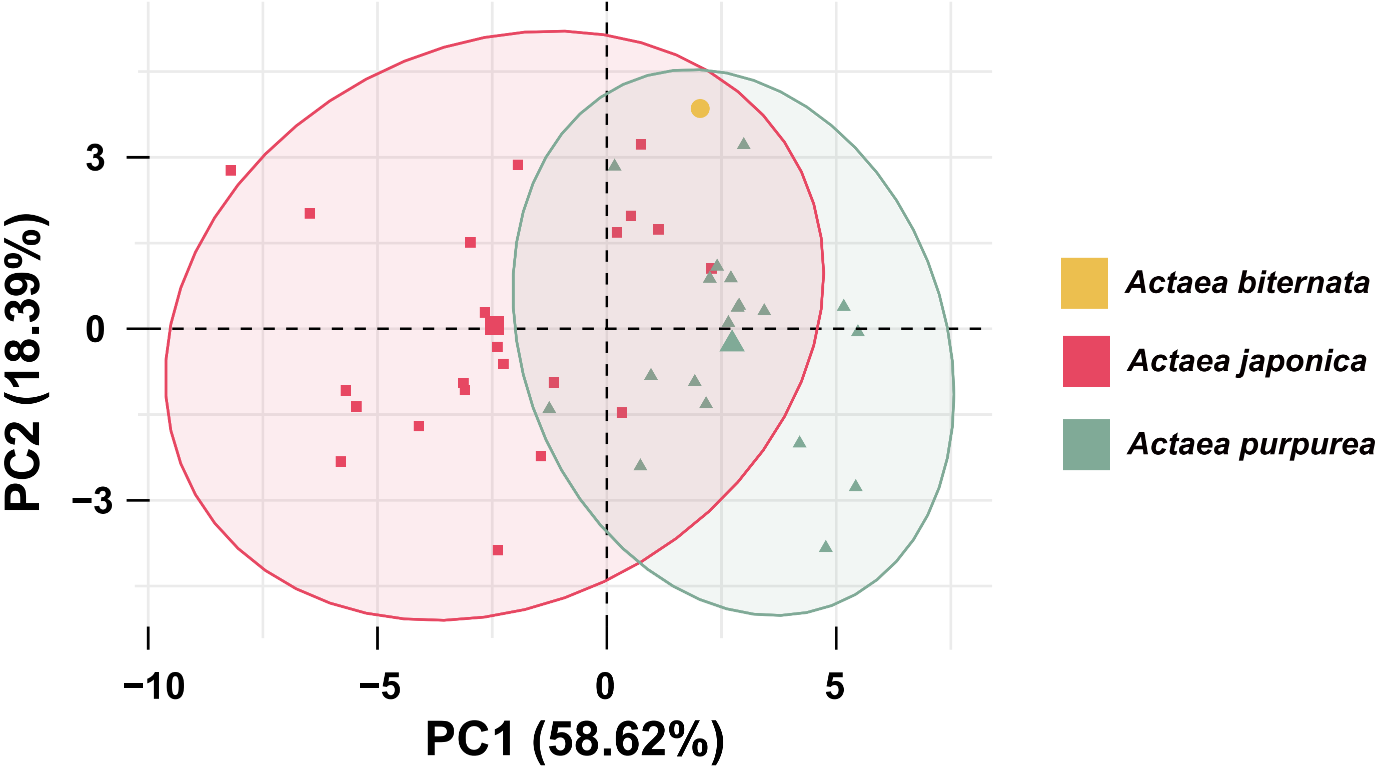

3
0
−3
−10
−5
0
5
PC1 (58.62%)
PC2 (18.39%)
Actaea biternata
Actaea japonica
Actaea purpurea
