# Supplementary material for: Nonadaptive molecular evolution of plastome during the speciation of Actaea purpurea and its relatives
Source: Ecol Evol. 2022 Sep 17;12(9):e9321. doi: 10.1002/ece3.9321 (PMC9482002; doi:10.1002/ece3.9321)
Supplement: Supplementary file 2 — Table S1–S5 [file ECE3-12-e9321-s002.docx]

**Table S1** Sampling information of *Actaea purpurea* and its relatives for plastome sequencing

| **Species** | **Population** | **Collecting locations** | **Longitude** | **Latitude** | **Voucher** | **GenBank** |
| --- | --- | --- | --- | --- | --- | --- |
| *Actaea biternata* | JP01 | Tokugawa, Saitama Prefecture, Japan | E 139°11'36" | N 35°59'28" | *Chang Xiao-Peng JP01* | OM460061 |
| *Actaea japonica* | GZ01 | Ziyun County, Guizhou, China | E 106°05'53" | N 25°51'26" | *Zhang Yuan-Zhen GZ01* | OM460062 |
|  | HB11 | Badong County, Hubei, China | E 110°14'56" | N 30°48'40" | *Chang Xiao-Peng HB11* | OM460063 |
|  | JJZ | Jinzhai County, Anhui, China | E 115°41'25" | N 31°13'01" | *Han Meng JJZ* | OM460064 |
|  | JP02 | Igawa Machi, Nagano Prefecture, Japan | E 138°40'20" | N 36°17'52" | *Chang Xiao-Peng JP02* | OM460065 |
|  | SC02 | Pingba village, Sichuan, China | E 102°34'50" | N 29°07'59." | *Zhang Yuan-Zhen SC02* | OM460066 |
|  | ZJ02 | Kuocang mountain, Zhejiang, China | E 120°54'57" | N 28°47'58" | *Chang Xiao-Peng ZJ02* | OM460067 |
| *Actaea purpurea* | HB01 | Shennongjia, Hubei Province, China | E 110°23'16" | N 31°28'10" | *Chang Xiao-Peng HB01* | OM460068 |
|  | HB04 | Dalaoling, Hubei, China | E 110°55'48" | N 31°04'19" | *Chang Xiao-Peng HB04* | OM460069 |
|  | HE02 | Pingshan, Hebei, China | E 113°48'23" | N 38°40'44" | *Xue Cheng & Niu Cheng-Yu HE02* | OM460070 |
|  | PJZ | Jinzhai County, Anhui, China | E 115°40'24" | N 31°14'57" | *Han Meng PJZ* | OM460071 |
|  | PZX | Zhuxi County, Hubei, China | E 109°41'26" | N 31°59'04" | *Zhong Dalv PZX* | OM460072 |
|  | SC01 | Laohegou nature reserve, Sichuan, China | E 104°43'01" | N 32°30'39" | *Zhang Yuan-Zhen SC01* | OM460073 |

**Table S2** List of genes in the plastomes of A. purpurea and its relatives

| **Category** | **Functional group** | **Genes** |
| --- | --- | --- |
| Photosynthesis related genes | Rubisco | *rbcL* |
|  | PhotosystemⅠ | *psaA, psaB, psaC, psaI, psaJ* |
|  | Assembly/stability of PhotosystemⅠ | *** ycf3* |
|  | Photosystem Ⅱ | *psbA, psbB, psbC, psbD, psbE, psbF, psbH, psbI, psbJ, psbK, psbL, psbM, psbN, psbT, psbZ* |
|  | ATP synthase | *atpA, atpB, atpE, * atpF, atpH, atpI* |
|  | cytochrome b/f complex | *petA, * petB, * petD, petG, petL, petN* |
|  | cytochrome c synthesis | *ccsA* |
|  | NADH dehydrogenase | * ndhA, * ndhB(×2), ndhC, ndhD, ndhE, ndhF, ndhG, ndhH, ndhI, ndhJ, ndhK |
| Transcription and translation related genes | transcription | *rpoA, rpoB, * rpoC1, rpoC2* |
|  | ribosomal proteins | rps2, rps3, rps4, rps7(×2), rps8, rps11, ** rps12(×2), rps14, rps15, * rps16, rps18, rps19, * rpl2(×2), rpl14, * rpl16, rpl20, rpl22, rpl23(×2), rpl33, rpl36 |
|  | translation initiation factor | *infA* |
| RNA genes | ribosomal RNA | rrn4.5(×2), rrn5(×2), rrn16(×2), rrn23(×2) |
|  | transfer RNA | trnA-UGC(×2), trnC-GCA, trnD-GUC, trnE-UUC, trnF-GAA, trnfM-CAU, trnG-GCC, trnG-UCC, trnH-GUG, = trnI-CAU(×2), trnI-GAU(×2), trnK-UUU, trnL-CAA(×2), trnL-UAA, trnL-UAG, trnM-CAU, trnN-GUU(×2), trnP-UGG, trnQ-UUG, trnR-ACG(×2), trnR-UCU, trnS-GCU, trnS-GGA, trnS-UGA, trnT-GGU, trnT-UGU, trnV-GAC(×2), trnV-UAC, trnW-CCA, trnY-GUA |
|  |  |  |
|  |  |  |
|  |  |  |
|  |  |  |
|  |  |  |
|  |  |  |
|  |  |  |
| Other genes | RNA processing | *matK* |
|  | carbon metabolism | *cemA* |
|  | fatty acid synthesis | *accD* |
|  | proteolysis | *** clpP* |
| Genes of unknown function | conserved reading frames | ycf1, ycf2(×2), ycf4 |

×2 means genes with two copies；*genes with one intron；**genes with two introns.

**Table S3** Sampling information of the 42 [sampling](javascript:;) [site](javascript:;)s used in the environmental analysis

| **Species** | **Collecting locations** | **Longitude** | **Latitude** | **Altitude (m)** | **Voucher** |
| --- | --- | --- | --- | --- | --- |
| *Actaea biternata* | Tokugawa, Saitama, Japan | E 139°11'36" | N 35°59′28″ | 500 | *Chang Xiao-Peng JP01* |
| *Actaea japonica* | Jinzai County, Anhui, China | E 115°45′48″ | N 31°07′18″ | 1590 | *Chang Xiao-Peng AH01* |
|  | Jinzai County, Anhui, China | E 115°44′20″ | N 31°16′31″ | 1575 | *Chang Xiao-Peng AH02* |
|  | Huangshan City, Anhui, China | E 118°09′57″ | N 30°08′47″ | 1570 | *Chang Xiao-Peng AH03* |
|  | Jinfo Mountain, Chongqing, China | E 107°11′21″ | N 28°57′50″ | 1230 | *Zhang Yuan-Zhen CQ01* |
|  | Ziyun County, Guizhou, China | E 106°05′53″ | N 25°51′26″ | 1600 | *Zhang Yuan-Zhen GZ01* |
|  | Badong County, Hubei, China | E 110°14′56″ | N 30°48′40″ | 1600 | *Chang Xiao-Peng HB11* |
|  | Tianping Mountain, Hunan, China | E 110°03′45″ | N 29°45′47″ | 1380 | *Chang Xiao-Peng HN01* |
|  | Ziyun Mountain, Hunan, China | E 111°05′49″ | N 26°36′23″ | 1200 | *Zhang Yuqv HN02* |
|  | Enshi City, Hubei, China | E 109°18′36″ | N 30°28′34″ | 1876 | *Zhang Yuan-Zhen JDS* |
|  | Huoshan County, Anhui, China | E 115°40′24″ | N 31°14′57″ | 1244 | *Han Meng JHS* |
|  | Jinzai County, Anhui, China | E 115°41′25″ | N 31°13′11″ | 900 | *Han Meng JJZ* |
|  | Enshi City, Hubei, China | E 108°57′51″ | N 29°22′49″ | 1433 | *Zhong Dalv JNS* |
|  | Igawa Machi, Nagano, Japan | E 138°40′20″ | N 36°17′52″ | 770 | *Chang Xiao-Peng JP02* |
|  | Shimen County, Hunan, China | E 110°33′16″ | N 30°01′45″ | 1236 | *Zhang Yuan-Zhen JSM* |
|  | Wuning County, Jiangxi, China | E 114°56′50″ | N 28°54′19″ | 1450 | *Chang Xiao-Peng JX01* |
|  | Pingba Village, Sichuan, China | E 102°34′50″ | N 29°07′59″ | 1820 | *Zhang Yuan-Zhen PSC* |
|  | Tianmu Mountain, Zhejiang, China | E 119°26′08″ | N 30°21′12″ | 1460 | *Chang Xiao-Peng & Zhang Yuqv ZJ01* |
|  | Linhai City, Zhejiang, China | E 120°54′57″ | N 28°47′58″ | 1050 | *Chang Xiao-Peng ZJ02* |
|  | Jiulong Mountain, Zhejiang, China | E 118°52′02″ | N 28°20′44″ | 1500 | *Chang Xiao-Peng ZJ03* |
| *Actaea purpurea* | Zhouqu County, Gansu, China | E 104°19′39″ | N 33°35′50″ | 1810 | *Zhang Yuan-Zhen GS01* |
|  | Laojun Mountain, Henan, China | E 111°38′02″ | N 33°43′09″ | 1700 | *Chang Xiao-Peng & Xue Cheng HA01* |
|  | Lingbao City, Henan, China | E 110°29′58″ | N 34°25′09″ | 1680 | *Xue Cheng & Niu Chenyu HA02* |
|  | Shennongjia, Hubei Province, China | E 110°23′16″ | N 31°28′10″ | 1270 | *Chang Xiao-Peng HB01* |
|  | Shennongjia, Hubei Province, China | E 110°25′34″ | N 31°40′29″ | 1400 | *Chang Xiao-Peng HB02* |
|  | Shennongjia, Hubei Province, China | E 110°13′57″ | N 31°37′34″ | 1300 | *Chang Xiao-Peng HB03* |
|  | Dalaoling, Hubei, China | E 110°55′48″ | N 31°04′19″ | 1700 | *Chang Xiao-Peng HB04* |
|  | Wu'an City, Hebei, China | E 113°46′15″ | N 36°56′10″ | 1300 | *Chang Xiao-Peng & Xue Cheng HE01* |
|  | Pingshan, Hebei, China | E 113°48′23″ | N 38°40′44″ | 1160 | *Xue Cheng & Niu Chenyu HE02* |
|  | Chengkou County, Chongqing, China | E 108°48′56″ | N 32°01′40″ | 1958 | *Zhang Yuan-Zhen & Zhong Dalv PBP* |
|  | Lushi County, Henan, China | E 110°49′44″ | N 33°44′58″ | 1545 | *Niu Chenyu & Han Meng PHA* |
|  | Jinzai County, Anhui, China | E 115°40′24″ | N 31°14′57″ | 1112 | *Han Meng PJZ* |
|  | Song County, Henan, China | E 111°59′50″ | N 33°41′21″ | 1140 | *Niu Chenyu & Han Meng PLC* |
|  | Luoyang City, Henan, China | E 111°25′50″ | N 34°08′52″ | 1701 | *Niu Chenyu & Han Meng PLN* |
|  | Xingshan County, Hubei, China | E 110°34′52″ | N 31°17′32″ | 1187 | *Zhang Yuan-Zhen PXS* |
|  | Zhuxi County, Hubei, China | E 109°41′26″ | N 31°59′04″ | 973 | *Zhong Dalv PZX* |
|  | Pingwu City, Sichuan, China | E 104°43′01″ | N 32°30′39″ | 1600 | *Zhang Yuan-Zhen SC01* |
|  | Liuba County, Shaanxi, China | E 106°49′58″ | N 33°40′22″ | 1370 | *Zhang Yuan-Zhen SN01* |
|  | Ningshan County, Shaanxi, China | E 108°30′02″ | N 33°33′41″ | 1490 | *Xue Cheng & Geng Fang-Dong SN02* |
|  | Long County, Shaanxi, China | E 106°32′32″ | N 35°04′03″ | 1900 | *Zhang Yuan-Zhen SN03* |
|  | Langao County, Shaanxi, China | E 109°04′08″ | N 32°14′42″ | 1890 | *Xue Cheng & Zhang Yuan-Zhen SN11* |
|  | Qinshui County, Shanxi, China | E 111°59′37″ | N 35°25′34″ | 1700 | *Xue Cheng & Niu Chenyu SX01* |

**Table S4** Principal component analysis results based on the 19 bioclimatic variables and altitude of *A. purpurea* and its relatives

| [**Environment**](javascript:;) [**Variable**](javascript:;) | **PC1 (58.62%)** | **PC2 (18.39%)** | **PC3 (13.05%)** |
| --- | --- | --- | --- |
| BIO01 = Annual mean temperature (°C*10) | 0.8040 | 0.4579 | -0.3489 |
| BIO02 = Mean diurnal range (Mean of monthly (max temp - min temp))(°C*10) | -0.7754 | 0.4243 | 0.1343 |
| BIO03 = Isothermality (BIO02/BIO07) (*100) | -0.5620 | 0.2199 | -0.4266 |
| BIO04 = Temperature seasonality (standard deviation *100) | -0.5116 | 0.3858 | 0.7387 |
| BIO05 = Max temperature of warmest month (°C*10) | 0.4168 | 0.8845 | 0.0446 |
| BIO06 = Min temperature of coldest month (°C*10) | 0.8601 | 0.0730 | -0.4897 |
| BIO07 = Temperature annual range (BIO05-BIO06) (°C*10) | -0.6842 | 0.4508 | 0.5585 |
| BIO08 = Mean temperature of wettest quarter (°C*10) | 0.3316 | 0.7881 | -0.0655 |
| BIO09 = Mean temperature of driest quarter (°C*10) | 0.8953 | 0.1069 | -0.3824 |
| BIO10 = Mean temperature of warmest quarter (°C*10) | 0.6342 | 0.7405 | 0.0249 |
| BIO11 = Mean temperature of coldest quarter (°C*10) | 0.822 | 0.2079 | -0.5230 |
| BIO12 = Annual precipitation (mm) | 0.9353 | -0.1944 | 0.2544 |
| BIO13 = Precipitation of wettest month (mm) | 0.8704 | -0.1855 | 0.3061 |
| BIO14 = Precipitation of driest month (mm) | 0.9175 | -0.1265 | 0.3324 |
| BIO15 = Precipitation seasonality (Coefficient of Variation) (mm) | -0.8669 | 0.1132 | -0.0689 |
| BIO16 = Precipitation of wettest quarter (mm) | 0.9167 | -0.1999 | 0.2406 |
| BIO17 = Precipitation of driest quarter (mm) | 0.8909 | -0.1150 | 0.3990 |
| BIO18 = Precipitation of warmest quarter (mm) | 0.8862 | -0.1550 | 0.2327 |
| BIO19 = Precipitation of coldest quarter (mm) | 0.8761 | -0.1554 | 0.4075 |
| BIO20 = [Altitude](javascript:;)（m） | -0.3385 | -0.8210 | -0.2294 |

**Table S5** Significant tests of differences of the 19 bioclimatic variables and altitude between *A. purpurea* and *A. japonica* + *A. biternata*

| [**Environment**](javascript:;) [**Variable**](javascript:;) | **Shapiro-Wilk’s test** | | **Welch’s two sample t test** | | **Wilcoxon rank sum test** | |
| --- | --- | --- | --- | --- | --- | --- |
|  | ***w*** | ***p*-vaule** | ***t*** | ***p*-vaule** | ***w*** | ***p*-vaule** |
| BIO01 = Annual mean temperature (°C*10) | 0.96936 | 3.14×10^-1^ | 4.55 | 5.53×10^-05^ |  |  |
| BIO02 = Mean diurnal range (Mean of monthly (max temp - min temp))(°C*10) | 0.91326 | **3.68×10^-3^** |  |  | 71.00 | 1.84×10^-4^ |
| BIO03 = Isothermality (BIO02/BIO07) (*100) | 0.93147 | **1.45×10^-2^** |  |  | 99.50 | 2.51×10^-3^ |
| BIO04 = Temperature seasonality (standard deviation *100) | 0.89754 | **1.22×10^-3^** |  |  | 123.50 | 1.56×10^-2^ |
| BIO05 = Max temperature of warmest month (°C*10) | 0.97262 | 4.03×10^-1^ | **1.96** | **5.65×10^-2^** |  |  |
| BIO06 = Min temperature of coldest month (°C*10) | 0.94814 | 5.54×10^-2^ | 5.00 | 1.67×10^-5^ |  |  |
| BIO07 = Temperature annual range (BIO05-BIO06) (°C*10) | 0.90088 | **1.53×10^-3^** |  |  | 81.50 | 5.06×10^-4^ |
| BIO08 = Mean temperature of wettest quarter (°C*10) | 0.97230 | 3.93×10^-1^ | **1.26** | **2.16×10^-1^** |  |  |
| BIO09 = Mean temperature of driest quarter (°C*10) | 0.96957 | 3.19×10^-1^ | 6.00 | 7.11×10^-7^ |  |  |
| BIO10 = Mean temperature of warmest quarter (°C*10) | 0.96286 | 1.87×10^-1^ | 3.02 | 4.40×10^-3^ |  |  |
| BIO11 = Mean temperature of coldest quarter (°C*10) | 0.96811 | 2.85×10^-1^ | 4.84 | 2.36×10^-5^ |  |  |
| BIO12 = Annual precipitation (mm) | 0.96725 | 2.66×10^-1^ | 6.73 | 5.00×10^-8^ |  |  |
| BIO13 = Precipitation of wettest month (mm) | 0.95627 | 1.09×10^-1^ | 5.68 | 2.16×10^-6^ |  |  |
| BIO14 = Precipitation of driest month (mm) | 0.94052 | **2.98×10^-2^** |  |  | 417.50 | 6.92×10^-7^ |
| BIO15 = Precipitation seasonality (Coefficient of Variation) (mm) | 0.86346 | **1.38×10^-4^** |  |  | 41.50 | 7.36×10^-6^ |
| BIO16 = Precipitation of wettest quarter (mm) | 0.96956 | 3.19×10^-1^ | 7.12 | 1.66×10^-8^ |  |  |
| BIO17 = Precipitation of driest quarter (mm) | 0.91966 | **5.89×10^-3^** |  |  | 414.5 | 1.03×10^-6^ |
| BIO18 = Precipitation of warmest quarter (mm) | 0.97279 | 4.08×10^-1^ | 6.92 | 2.49×10^-8^ |  |  |
| BIO19 = Precipitation of coldest quarter (mm) | 0.90799 | **2.52×10^-3^** |  |  | 414.50 | 1.03×10^-6^ |
| BIO20 = [Altitude](javascript:;)（m） | 0.97269 | 4.05×10^-1^ | **-1.49** | **1.46×10^-1^** |  |  |

In the Shapiro-Wilk’s test, significant values are in bold; In the other two tests, non-significant values are in bold
